# The role of 3-ketosteroid 1(2)-dehydrogenase in the pathogenicity of *Mycobacterium tuberculosis*

**DOI:** 10.1186/1471-2180-13-43

**Published:** 2013-02-20

**Authors:** Marta Brzezinska, Izabela Szulc, Anna Brzostek, Magdalena Klink, Michal Kielbik, Zofia Sulowska, Jakub Pawelczyk, Jaroslaw Dziadek

**Affiliations:** 1 Institute of Medical Biology, Polish Academy of Sciences, Lodowa 106 93–232, Lodz, Poland

**Keywords:** *Mycobacterium tuberculosis*, Human macrophages, Ketosteroid dehydrogenase, Bactericidal activity, TLR2 signaling pathway

## Abstract

**Background:**

A growing body of evidence suggests that *Mycobacterium tuberculosis* (Mtb) uses the host’s cholesterol as a source of carbon and energy during infection. Strains defective in cholesterol transport or degradation exhibit attenuated growth in activated macrophages and diminished infectivity in animal models. The aim of this study was to evaluate intracellular replication of a cholesterol degradation-deficient Mtb mutant in human macrophages (MØ) *in vitro* and assess the functional responses of Mtb mutant-infected MØ.

**Results:**

A mutant Mtb H37Rv strain containing an inactivated *kstD* gene (∆*kstD*), which encodes 3-ketosteroid 1(2)-dehydrogenase (KstD), was previously prepared using the homologous recombination-based gene-replacement technique. A control strain carrying the *kstD* gene complemented with an intact *kstD* was also previously constructed. In this study, human resting MØ were obtained after overnight differentiation of the human monocyte-macrophage cell line THP-1. Resting MØ were further activated with interferon-γ (IFN-γ). The ability of the *kstD*-defective Mtb mutant strain to replicate intracellularly in human MØ was evaluated using a colony-forming assay. Nitric oxide (NO) and reactive oxygen species (ROS) production by MØ infected with wild-type or ∆*kstD* strains was detected using Griess reagent and chemiluminescence methods, respectively. The production of tumor necrosis factor-α and interleukin-10 by MØ after infection with wild-type or mutant Mtb was examined using enzyme-linked immunosorbent assays.

We found that replication of mutant Mtb was attenuated in resting MØ compared to the wild-type or complemented strains. Moreover, the mutant was unable to inhibit the NO and ROS production induced through Toll-like receptor 2 (TLR2) signaling in infected resting MØ. In contrast, mutant and wild-type Mtb behaved similarly in MØ activated with IFN-γ before and during infection.

**Conclusions:**

The Mtb mutant ∆*kstD* strain, which is unable to use cholesterol as a source of carbon and energy, has a limited ability to multiply in resting MØ following infection, reflecting a failure of the ∆*kstD* strain to inhibit the TLR2-dependent bactericidal activity of resting MØ.

## Background

Alveolar macrophages (MØ) represent the host’s first line of defense against *Mycobacterium tuberculosis* (Mtb). Phagocytosed Mtb bacilli are subjected to degradation via oxygen-dependent and -independent mechanisms. In the oxygen-dependent mechanism, MØ produce a variety of powerful mediators such as reactive oxygen species (ROS) and reactive nitrogen intermediates (RNI) that kill bacteria [1,2].

The first step in the activation of innate host defenses against Mtb is the recognition of the pathogen. Host receptors involved in bacterial recognition and phagocytosis include complement receptors and pattern recognition receptors. Among the latter category are Toll-like receptors (TLRs), which mediate stimulation of the immune cells. TLR2, in particular, is known to be involved in the recognition of Mtb. After interaction of a specific structure of the mycobacterial cell wall with TLR2, a signaling pathway cascade is initiated in which interleukin 1 receptor associated kinase-1 and −4 (IRAK-1/4) associate with TLR2 via the adaptor protein MyD88. IRAK-1/4 then phosphorylate and activate the protein TRAF-6 (tumor necrosis factor receptor-associated factor-6), which in turn activates other signaling proteins, including mitogen-activated protein kinases (MAPKs), phosphoinositide 3-kinase, protein kinase C, and nuclear factor κB. This leads to the transcription of genes involved in the production of nitric oxide (NO) and various cytokines, such as interleukin (IL)-1β, tumor necrosis factor-α (TNF-α), IL-10 and IL-12, and promotes activation of the NADPH oxidase complex, which is responsible for ROS production [2]-2 [7]. In the context of initial infection, MØ encounters Mtb prior to being stimulated with the Th1 cytokine interferon-γ (IFN-γ). However, full activation of MØ antimicrobial capacity and antigen-presentation function only occurs after stimulation with IFN-γ [8].

During infection, Mtb adapts to different nutrient conditions to utilize fatty acids, which are alternative carbon and energy sources for tubercle bacilli. It is generally accepted that Mtb can use cholesterol as a source of carbon and energy. The full suite of genes required for cholesterol degradation has been identified in the Mtb genome, and the inactivation of cholesterol uptake by disruption of the ABC-like transport system has been shown to affect cholesterol degradation [9]. A similar effect was observed following disruption of 3-ketosteroid 1 (2)-dehydrogenase (KstD), 3-ketosteroid 9OH-hydroxylase (KshA/KshB), and the iron-dependent extradiol dioxygenase (HsaC) key enzymes involved in opening the steroid ring structure [10-12]. We have previously shown that tubercle bacilli can accumulate cholesterol in the free-lipid zone of their cell walls [10]. We have also demonstrated that Mtb utilizes cholesterol via the androstenedione/androstadienedione pathway (AD/ADD) using KstD, which initiates steroid ring degradation through transhydrogenation of 3-keto-4-ene steroids to 3-keto-1,4-diene steroids and that KstD is an essential enzyme in this process [10,13]. The Mtb ∆*kstD* strain lacking functional KstD accumulates non-toxic cholesterol degradation intermediates, AD and 9OHAD (9a-hydroxy-4-androstene-3,17-dione) [10], and is unable to grow on minimal medium supplemented with cholesterol as a sole carbon and energy source. However, the relationship between the altered growth of the ∆*kstD* mutant strain and the possible attenuation of the infection process has not been previously described.

Here, we evaluated the ability of an Mtb strain lacking a functional copy of the *kstD* gene to grow in human MØ. The functional activity of MØ against the parental Mtb H37Rv strain and the ∆*kstD* mutant strain was compared by examining the production of NO, ROS, TNF-α and IL-10 by resting and IFN-γ-activated MØ.

## Methods

### Chemicals and antibodies

RPMI-1640 medium containing 1 mM sodium pyruvate, Dulbecco’s phosphate-buffered saline (D-PBS) and Hanks’ balanced salt solution (HBSS) were purchased from Gibco (Scotland). Middlebrook OADC (oleic acid albumin dextrose catalase) enrichment, Middlebrook 7H9 broth, and Middlebrook 7H10 agar were obtained from Becton Dickinson (USA). IFN-γ, phorbol 12-myristate 13-acetate (PMA), bovine serum albumin (BSA), fluorescein isothiocyanate (FITC), Tween-20, Tween-80, IRAK1/4 inhibitor, 37% formaldehyde solution (FA), horseradish peroxidase (HRP), 2-mercaptoethanol (2-ME) and luminol were purchased from Sigma-Aldrich (USA). Human type AB serum (off-clot) and fetal bovine serum (FBS) were purchased from PAA-The Cell Culture Company (Austria). Mouse IgG_2a_ anti-human TLR2 (sodium azide-free), phycoerythrin (PE)-conjugated mouse anti-TLR2 (IgG_2a_), and PE-conjugated mouse IgG_2aκ_ isotype control were obtained from Imgenex (USA). FITC-conjugated mouse anti-human CD14 (IgG_2aκ_) and PE-conjugated anti-human CD11b (IgG_1κ_) were purchased from BD Pharmingen (USA). Human TNF-α and human IL-10 Quantikine enzyme-linked immunosorbent assay (ELISA) kits were purchased from R&D Systems (USA).

### Bacterial strains and growth conditions

All strains used in this study were based on *M. tuberculosis* H37Rv (ATCC) and were maintained on Middlebrook 7H10 agar or 7H9 broth supplemented with 10% OADC enrichment and 25 μg/ml kanamycin, as required. For growth on media supplemented with defined carbon sources, strains were grown in minimal medium supplemented with 0.01% cholesterol, as described previously [9]. The engineering of the Mtb strain deficient for the KstD enzyme (Δ*kstD*), and Δ*kstD* complemented with an intact *kstD* gene (Δ*kstD-kstD*) was described previously [10]. Wild-type, mutant, and complemented bacterial strains were prepared for infection by growing in roller bottles in Middlebrook 7H9 broth containing 10% OADC enrichment and 0.05% Tween-80 for 4–6 days to reach an optical density at 600 nm (OD_600_) of 1. A portion of the bacterial culture (approximately 1 × 10^9^ bacilli/ml) was suspended in Middlebrook 7H9 broth and labeled with 100 μg/ml of FITC by incubating for 2 hours at room temperature with gentle agitation in the dark. FITC-labeled bacteria were washed once with Middlebrook 7H9 broth supplemented with 4% BSA and then twice with Middlebrook 7H9 broth without BSA. Unlabeled and FITC-labeled bacteria were divided into equal portions and stored at -85°C. After 1 week, a portion of bacteria was thawed and colony-forming assays were used to determine the number of bacterial colony-forming units (CFUs).

### Cells culture

The human monocyte-macrophages cell line THP-1 (ATCC TIB-202, USA) was maintained in culture medium (CM) consisting of RPMI-1640 supplemented with 1 mM sodium pyruvate, 10% FBS, 0.05 mM 2-ME, 100 U/ml of penicillin and 100 μg/ml of streptomycin at 37°C in a humidified 5% CO_2_ environment. THP-1 cells were passaged every 3–4 days.

Undifferentiated THP-1 cells (monocytes) were distributed into 24- and 96-well plates and differentiated into macrophages (resting MØ) by culturing for 24 hours (37°C, 5% CO_2_) with PMA (20 ng/ml), as described previously by others [14-16]. The macrophage-like phenotype of the cells was confirmed by assessing CD14 expression using flow cytometry (see below). The ability of resting MØ to adhere to plastic dishes was examined under a light microscope. IFN-γ-activated MØ were prepared by incubating resting MØ with 20 ng/ml of IFN-γ in CM for 24 hours (37°C, 5% CO_2_). Resting MØ and IFN-γ-activated MØ were infected with bacteria and cultured in CM without antibiotics. IFN-γ (20 ng/ml) was added to cultures of IFN-γ-activated MØ.

### Flow cytometry analysis

CD14 surface expression on monocytes and resting MØ was assessed by staining the cells (1 × 10^5^) with 10 μg/ml of a FITC-conjugated monoclonal antibody (mAb) against CD14 or isotype control (IgG_2a_; 10 μg/ml) for 30 minutes at 4°C. Before staining with anti-TLR2 mAb, crystallizable fragment receptors (FcRs) were blocked in D-PBS containing 10% human AB serum for 15 minutes at room temperature to prevent nonspecific antibody binding. Subsequently, cells were washed twice in D-PBS containing 1% FBS. Resting MØ and IFN-γ-activated MØ (1 × 10^5^ cells) were stained with 10 μg/ml of a PE-conjugated anti-TLR2 mAb or isotype control (IgG_1_; 10 μg/ml). A concentration of anti-TLR2 mAb sufficient to completely block the expression of TLR2 on cells was determined in preliminary experiments by adding different mAb concentrations (10, 25, and 35 μg/ml) to MØ and incubating for 1 hour (37°C/5% CO_2_). MØ were then stained with PE-conjugated anti-TLR2 mAb or isotype control, as described above.

All stained cells were washed twice, resuspended in 200 μl of D-PBS containing 1% FBS, 1% FA and sodium azide, and stored at 4°C until FACS (fluorescence-activated cell sorting) analysis. All samples were examined with a FACS LSR II BD flow cytometer (Becton Dickinson, USA) equipped with BD FACS Diva Software. The results were presented as median fluorescence intensity (MFI), which correlates with the surface expression of the target molecule.

### MØ infection

Bacteria were thawed, washed twice in RPMI-1640 medium, and then opsonized (or not) by incubating with 20% human serum AB in RPMI-1640 medium for 30 minutes at 37°C with gentle agitation. Thereafter, bacteria were washed once with RPMI-1640 medium. Opsonized and non-opsonized Mtb were suspended in CM, and clumps were disrupted by multiple passages through a 25-gauge needle. Serial dilutions of bacteria were prepared in CM.

Resting MØ and IFN-γ-activated MØ were infected with opsonized or non-opsonized, unlabeled or FITC-labeled Mtb strains (wild-type, mutant [Δ*kstD*] or complemented [∆*kstD- kstD*]) at a multiplicity of infection (MOI) of 1 or 10, as indicated, for 2 hours (37°C, 5% CO_2_). Extracellular bacteria were removed by extensively washing MØ with warm HBSS. Infected MØ were directly used in tests (day 0) or cultured for 1, 2 or 6 days, as indicated in Figures.

### Ingestion of bacteria

Resting MØ and IFN-γ-activated MØ (1 × 10^5^ cells/well) were prepared in 8-well Permanox chamber slides (Nunc, Denmark) and then infected with FITC-labeled Mtb strains at an MOI of 10. After infection, MØ were fixed by incubating with 3% FA for 15 minutes (37°C, 5%, CO_2_) and washed twice with HBSS. The number of infected MØ and the number of bacteria engulfed by one MØ were determined by fluorescence microscopic examination (Nikon ECLIPSE TE 2000 U). In all cases, 200 MØ were counted.

### Intracellular growth of bacteria

Resting MØ and IFN-γ-activated MØ (1 × 10^5^ cells/well) were prepared in 24-well plates (Nunc). MØ were then treated with 10 μM IRAK1/4 inhibitor or with a saturating concentration of anti-TLR2 blocking mAbs (35 μg/ml) for 1 hour or left untreated. Afterwards, MØ were infected with Mtb strains at an MOI of 1. After infection, fresh CM and IRAK1/4 inhibitor or anti-TLR2 blocking mAb (when required) were added, and cells were cultured for 6 days. On the day of infection (day 0) and 6 days post-infection, MØ were lysed with 1 ml of 0.2% Triton X-100 and appropriate dilutions of cell lysates were plated onto Middlebrook 7H10 agar supplemented with 10% OADC. After 21 days of culture, CFUs were counted. The data were presented as fold-increase in CFUs, calculated as CFUs on day 6 divided by CFUs on day 0.

### NO production

Resting MØ and IFN-γ-activated MØ (1 × 10^5^ cells/well) were prepared in 96-well plates (Nunc) and treated with IRAK1/4 inhibitor or left untreated (as described above). Next, MØ were infected with Mtb strains at an MOI of 10 and cultured for 2 days with or without IRAK1/4 inhibitor. The presence of nitrite (stable metabolite of NO) in the culture supernatants was determined using the Griess reagent. OD was determined using a Multiscan RC ELISA reader (Labsystem, Finland). Nitrite concentration was calculated from a standard curve prepared using sodium nitrite as a reference.

### ROS production

Resting MØ and IFN-γ-activated MØ (1 × 10^5^ cells/well) were prepared in 96-well plates (Nunc) and then infected with Mtb strains at an MOI of 10. After culturing for 1 day, 1 μg/ml of PMA (to initiate ROS production) as well as 40 U of HRP and 1 mM luminol (to enhance chemiluminescence) were added to the cells. Chemiluminescence (CL) was recorded over 4 hours at 5-minute intervals using Fluoroscan Ascent FL (Labsystem, Finland). Data were acquired as relative light units (RLUs), and the area under the curve of CL versus assay time (total RLUs) was calculated. Data were presented as percent inhibition of ROS production calculated according to the formula, 1 - (total RLUs for cells infected with bacteria and stimulated with PMA/total RLUs for cells stimulated with PMA) × 100.

### TNF-α and IL-10 production

Resting MØ and IFN-γ-activated MØ (1 × 10^6^ cells/well) were prepared in 24-well plates (Nunc), infected with Mtb strains (MOI of 10), and cultured for 24 hours. The presence of TNF-α and IL-10 in the culture supernatants was assessed using Quantikine ELISA kits. The sensitivities of TNF-α and IL-10 assays were 1.6 pg/ml and 3.9 pg/ml, respectively.

### Statistical analysis

Data are presented as means ± SEMs. Statistical significance was verified using nonparametric Wilcoxon’s signed-rank or Mann–Whitney *U* tests. The Statistica 8.0 (StatSoft, Poland) software package was used for statistical calculations. Statistical significance was defined as p ≤ 0.05.

## Results

### Expression of CD14 on resting MØ

In order to confirm that THP-1 cells in the presence of PMA were differentiated after 24 hours, the surface expression of CD14 molecule was estimated. Similarly to other researchers [17,18] we found that CD14 surface expression on monocytes (i.e., THP-1 cells prior to differentiation) was greater than on PMA-treated THP-1 cells (i.e., after differentiation to MØ), with MFI values of 99 ± 10 and 45 ± 7 (n = 6), respectively.

### MØ uptake of ∆kstD mutant and wild-type strains

**Figure 1:**
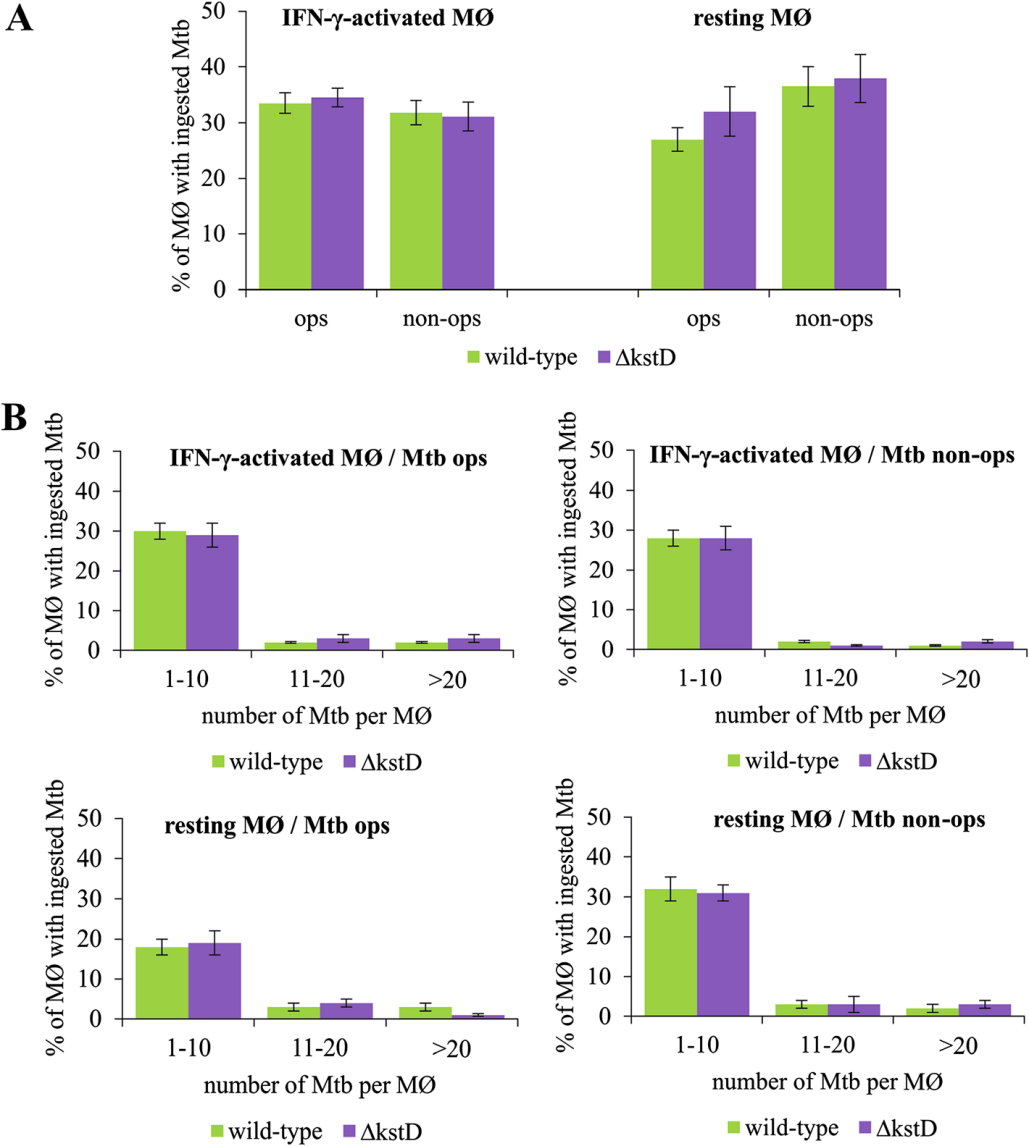
**Ingestion of Mtb by MØ.** Resting and IFN-γ-activated MØ were infected with FITC-labeled wild-type or ∆*kstD* strains for 2 hours. (**A**) Percentage of MØ infected with Mtb strains; (**B**) Percentage distribution of MØ with the counted number of bacteria engulfed by one phagocyte (per MØ). Percentage of infected MØ was calculated according to the formula: MØ with bacteria *100/ number of counted MØ and expressed as means ± SEMs (n = 5). Mtb ops – bacteria opsonized, Mtb non-ops – bacteria non-opsonized.

The percentage of resting MØ and IFN-γ-activated MØ involved in the uptake of Mtb strains was approximately 30-40%. Moreover, both types of MØ ingested opsonized and non-opsonized wild-type and *∆kstD* strains equally well (Figure 1A), and took up similar numbers of bacteria of both strains (Figure 1B).

### Intracellular replication of wild-type and ∆kstD strains

**Figure 2:**
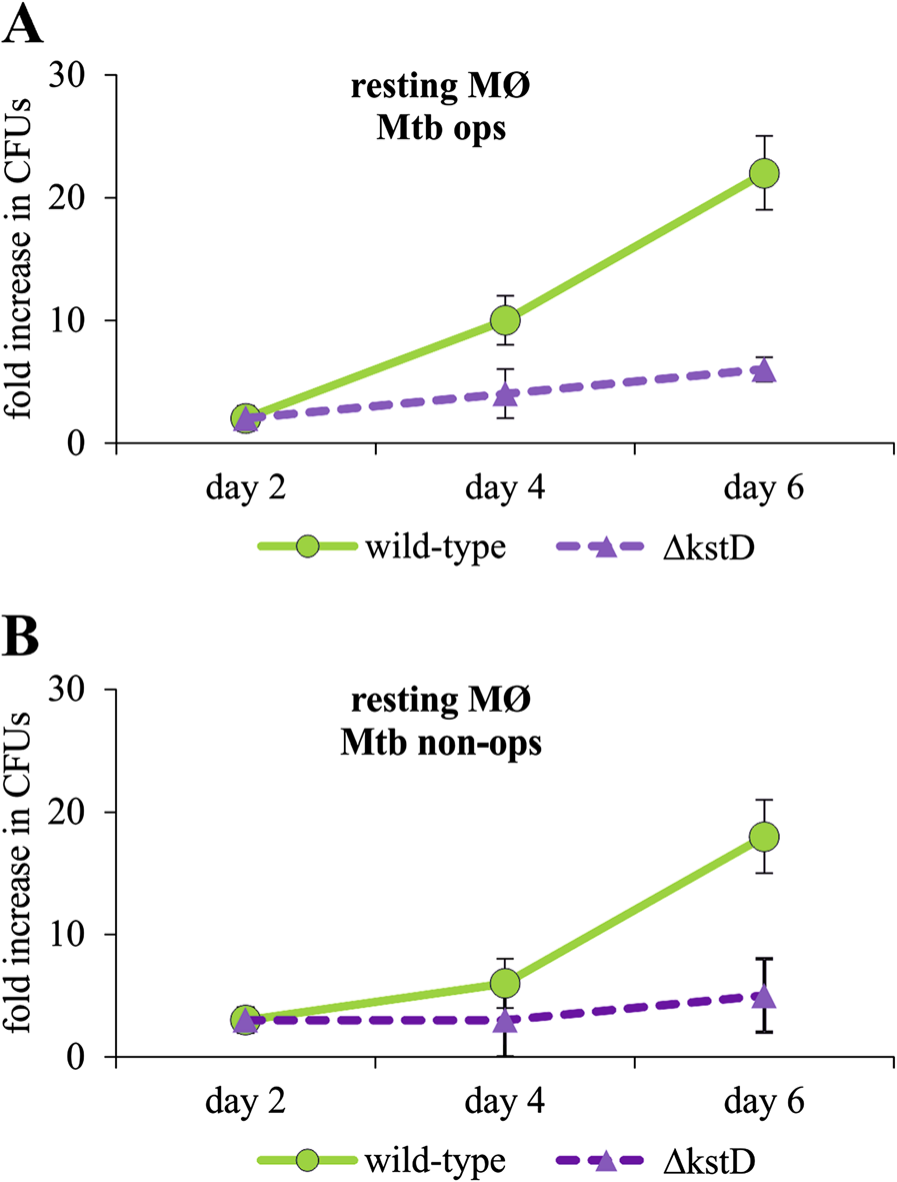
**Time-dependent survival of Mtb in MØ.** Resting MØ were infected with wild-type or ∆*kstD* strains for 2 hours. On the day of infection and after 2, 4 or 6 days in culture, MØ were lysed with Triton X-100 and cell lysates were plated onto agar plates. After 21 days of culture, CFUs/ml were counted. The data are presented as fold increase in CFUs/ml, expressed as means ± SEMs (n = 3). Mtb ops – bacteria opsonized, Mtb non-ops – bacteria non-opsonized.

**Figure 3:**
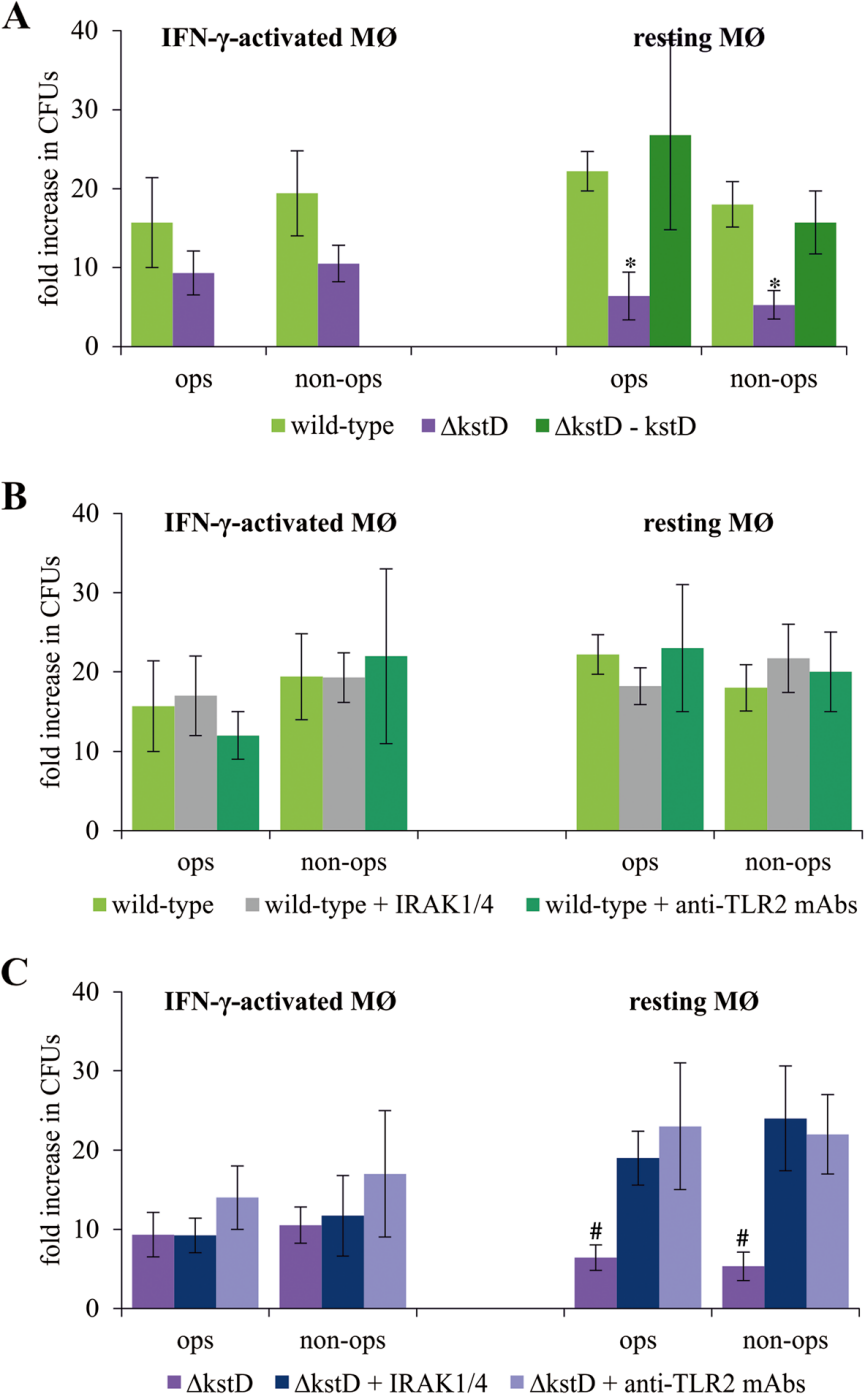
**Survival of Mtb in MØ.** (**A**) Resting MØ and IFN-γ-activated MØ were infected with wild-type, ∆*kstD*, or ∆*kstD*-*kstD* strains for 2 hours without inhibitors. Resting MØ were pre-incubated with IRAK1/4 inhibitor or anti-TLR2 blocking mAb for 1 hour prior to infection with wild-type (**B**) or ∆*kstD* (**C**) strains. On the day of infection and after 6 days in culture, MØ were lysed with Triton X-100 and cell lysates were plated onto agar plates. After 21 days of culture, CFUs/ml were counted. The data are presented as fold increase in CFUs/ml, expressed as means ± SEMs (n = 5; *p ≤ 0.05, ∆*kstD* vs. wild-type or ∆*kstD*-*kstD*; #p ≤ 0.02, ∆*kstD* vs. ∆*kstD* + IRAK1/4 or ∆*kstD* + anti-TLR2 mAb; Mann–Whitney *U* test). ops – bacteria opsonized, non-ops – bacteria non-opsonized.

Initially, we compared the survival of the wild-type and Δ*kstD* strains in resting MØ 1, 2, 4, 6 and 8 days post-infection. The detachment of MØ monolayer was observed on day 8 and therefore this time point was excluded from the subsequent experiments. We did not observe differences in CFUs count at 1 and 2 days post-infection, therefore day 1 was also excluded from the subsequent experiments. As shown in Figure 2, the numbers of viable wild-type and Δ*kstD* bacilli were similar up to 2 days post-infection, slightly and insignificantly different up to 4 days and statistically different on day 6, suggesting differential growth of mutant and wild-type strains. To test this, we compared the intracellular replication of Δ*kstD* and wild-type Mtb in resting and IFN-γ-activated MØ 6 days after infection. We found that survival of opsonized and non-opsonized wild-type and Δ*kstD* strains was slightly but insignificantly different in IFN-γ-activated MØ. In contrast, growth of mutant strain was significantly attenuated in resting MØ compared to wild-type and complemented (Δ*kstD*-*kstD*) strains (Figure 3A). It should also be noticed that the initial CFUs/ml values (day 0) for wild-type and mutant strains did not differ statistically. CFUs/ml values of opsonized and non-opsonized wild-type and Δ*kstD* strains for IFN-γ-activated MØ amounted: 1425 ± 507; 3270 ± 1715 and 2550 ± 845; 2150 ± 556, respectively and for resting MØ amounted: 1612 ± 412; 3140 ± 1330 and 1950 ± 1177; 2760 ± 1250, respectively.

We found that TLR2 expression level on resting MØ was higher than on monocytes and the treatment of MØ with IFN-γ enhanced this expression (MFI = 115 ± 7 and MFI = 71 ± 10, and MFI = 171 ± 13, respectively). By using flow cytometry we found that surface expression of TLR2 was virtually undetectable (MFI = 32 ± 5) after pre-incubation of resting MØ and IFN-γ-activated MØ with 35 μg/ml of blocking anti-TLR2 mAb.

The presence of IRAK1/4 inhibitor or anti-TLR2 blocking mAb insignificantly influenced the survival of the wild-type strain in either type of MØ (Figure 3B). In contrast, inhibition of the TLR2 signaling pathway significantly increased the growth of both opsonized and non-opsonized Δ*kstD* in resting MØ (Figure 3C). Dimethyl sulfoxide (DMSO), used as a vehicle to prepare IRAK1/4 inhibitor solutions, had no effect on the growth of Mtb strains in MØ at the final concentration present in CM containing IRAK1/4 inhibitor (0.5%) (data not shown).

### ROS and NO production by MØ infected with wild-type, Δ*kstD,* or Δ*kstD*-*kstD* strains

**Figure 4:**
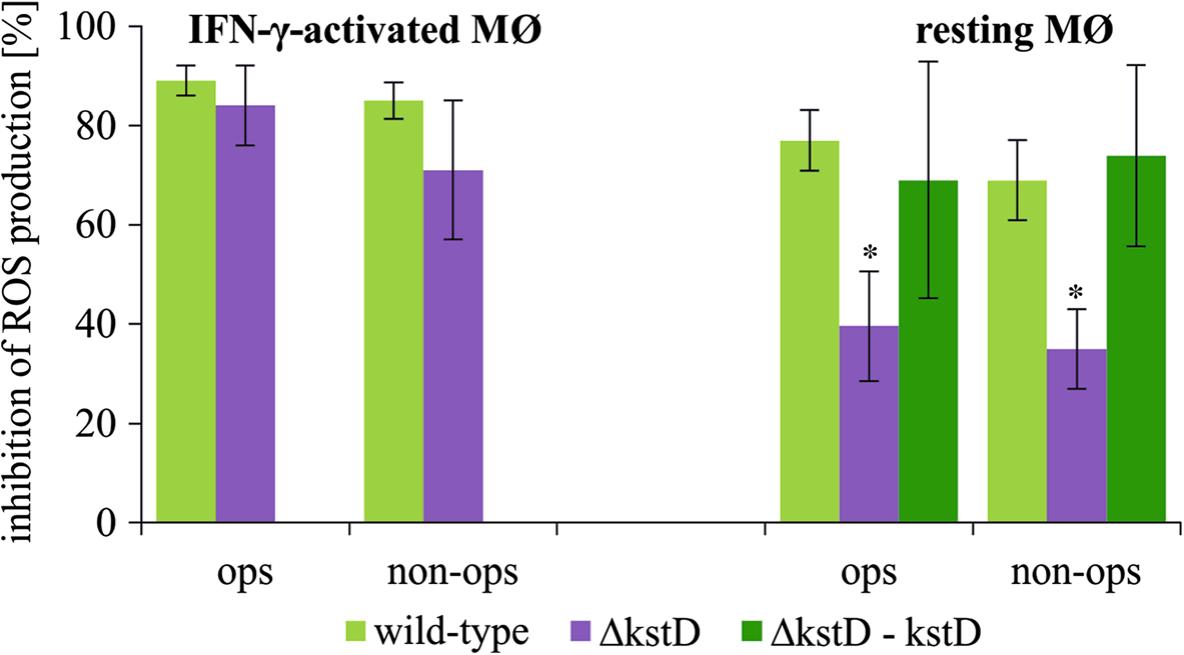
**ROS production by infected MØ.** Resting MØ and IFN-γ-activated MØ were infected with wild-type, ∆*kstD*, or ∆*kstD*-*kstD* strains for 2 hours and cultured for 1 day. Cells were then stimulated with PMA, and ROS production was assessed using the CL assay. Data are presented as the percentage of ROS production inhibition, expressed as means ± SEMs (n = 5; *p ≤ 0.04, ∆*kstD* vs. wild-type or ∆*kstD*-*kstD*; Mann–Whitney *U* test). ops – bacteria opsonized, non-ops – bacteria non-opsonized.

We next tested the influence of Mtb on spontaneous and PMA-stimulated ROS production by MØ 1 day post-infection. In a preliminary study, we noted that wild-type Mtb did not affect spontaneous ROS production by MØ, but strongly inhibited the ability of resting and IFN-γ-activated MØ to produce ROS in response to PMA activation (data not shown). Therefore, in subsequent experiments, we compared the effect of wild-type and Δ*kstD* mutant Mtb on ROS production by resting and IFN-γ-activated MØ in the presence of PMA. We found that the production of ROS in IFN-γ-activated MØ was inhibited to a similar extent by the *ΔkstD* mutant and the wild-type strain. However, opsonized and non-opsonized *ΔkstD* exhibited a significantly weaker ability to inhibit ROS production in resting MØ compared to wild-type and complemented strains (Figure 4). Two days post-infection wild-type Mtb had significantly higher ability to inhibit ROS production by resting MØ than mutant strain. The percentage of inhibition of ROS production induced with wild-type opsonized or non-opsonized and *ΔkstD* mutant opsonized or non-opsonized amounted: 78 ± 7; 40 ± 8 and 33 ± 22; 34 ± 14, respectively. Neither the vehicle control for PMA (0.1% ethanol in HBSS) nor 0.5% DMSO (in HBSS) affected ROS production by resting MØ (17 and 20 RLU for ethanol and DMSO solutions, respectively) or activated MØ (74 and 71 RLU for ethanol and DMSO solutions, respectively).

**Figure 5:**
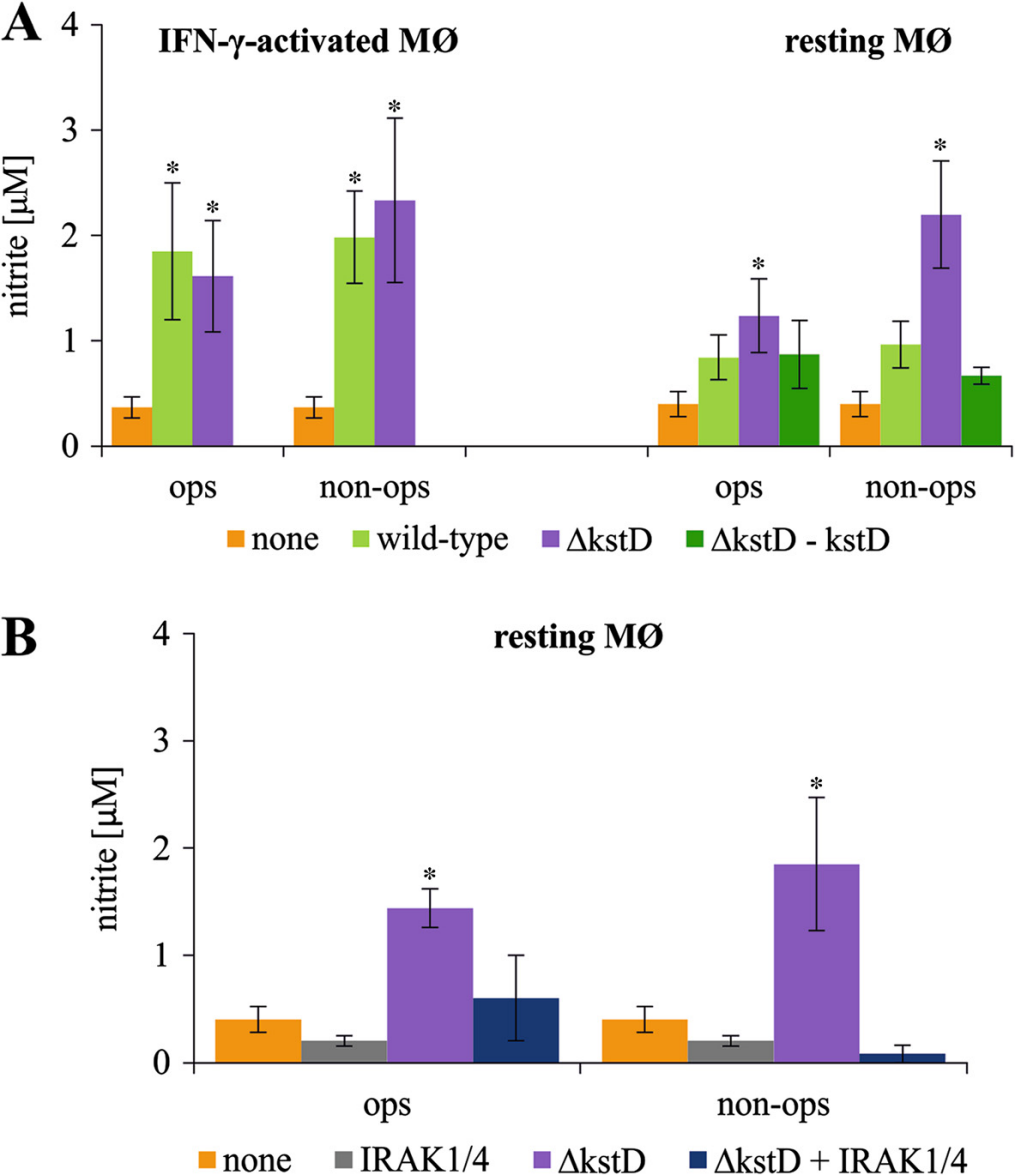
**NO production by infected MØ.** (**A**) Resting MØ and IFN-γ-activated MØ were infected with wild-type, ∆*kstD*, or ∆*kstD*-*kstD* strains for 2 hours without inhibitors. (**B**) Resting MØ were pre-incubated with IRAK1/4 inhibitor for 1 hour prior to infection with ∆*kstD.* After culturing for 2 days, the concentration of nitrite, a stable metabolite of NO, was assessed in culture supernatants using the Griess reagent. The data are presented as nitrite concentration, expressed as means (μM) ± SEMs (n = 6; *p ≤ 0.03, strain vs. none [MØ in CM]; Wilcoxon’s signed-rank test). ops – bacteria opsonized, non-ops – bacteria non-opsonized; none – MØ in culture medium (control).

Because preliminary experiments demonstrated that the level of nitrite (a stable metabolite of NO) was almost undetectable in culture supernatants 1 day after infection, NO production by MØ was determined on day 2 post-infection. We found no significant differences in the production of NO by IFN-γ-activated MØ (in which iNOS expression is initiated by IFN-γ) infected with wild-type or mutant strains. The level of nitrite in culture supernatants of IFN-γ-activated MØ amounted 0.33 ± 0.10 μM for uninfected MØ, 1.85 ± 0.65 μM and 1.98 ± 0.44 μM for phagocytes infected with wild-type opsonized and non-opsonized, respectively and 1.61 ± 0.59 μM and 2.33 ± 0.70 μM for phagocytes infected with Δ*kstD* strain opsonized and non-opsonized, respectively. In contrast, resting MØ produced significant amount of NO only after infection with non-opsonized and opsonized Δ*kstD* strain (Figure 5A). However, the difference observed in the production of NO by resting MØ treated with opsonized or non-opsonized Δ*kstD* mutant was statistically insignificant. The amount of nitrite in supernatants of uninfected resting MØ was 0.40 ± 0.12 μM, in supernatants of resting MØ infected with non-opsonized and opsonized wild-type Mtb was 0.84 ± 0.2 μM and 0.90 ± 0.22 μM, respectively, and infected with non-opsonized and opsonized mutant strain was 1,24 ± 0.35 and 2.20 ± 0.53 μM, respectively. Notably, NO production induced in mutant Mtb-infected MØ was attenuated by treatment with IRAK1/4 inhibitor (Figure 5B). As was the case for other parameters, DMSO (0.5%) had no effect on NO production by resting or IFN-γ-activated MØ (0.40 ± 0.2 μM vs. 0.37 ± 0.2 μM nitrite in the presence and absence of DMSO, respectively).

### TNF-α and IL-10 production by MØ infected with wild-type, ΔkstD, or ΔkstD-kstD strains

**Figure 6:**
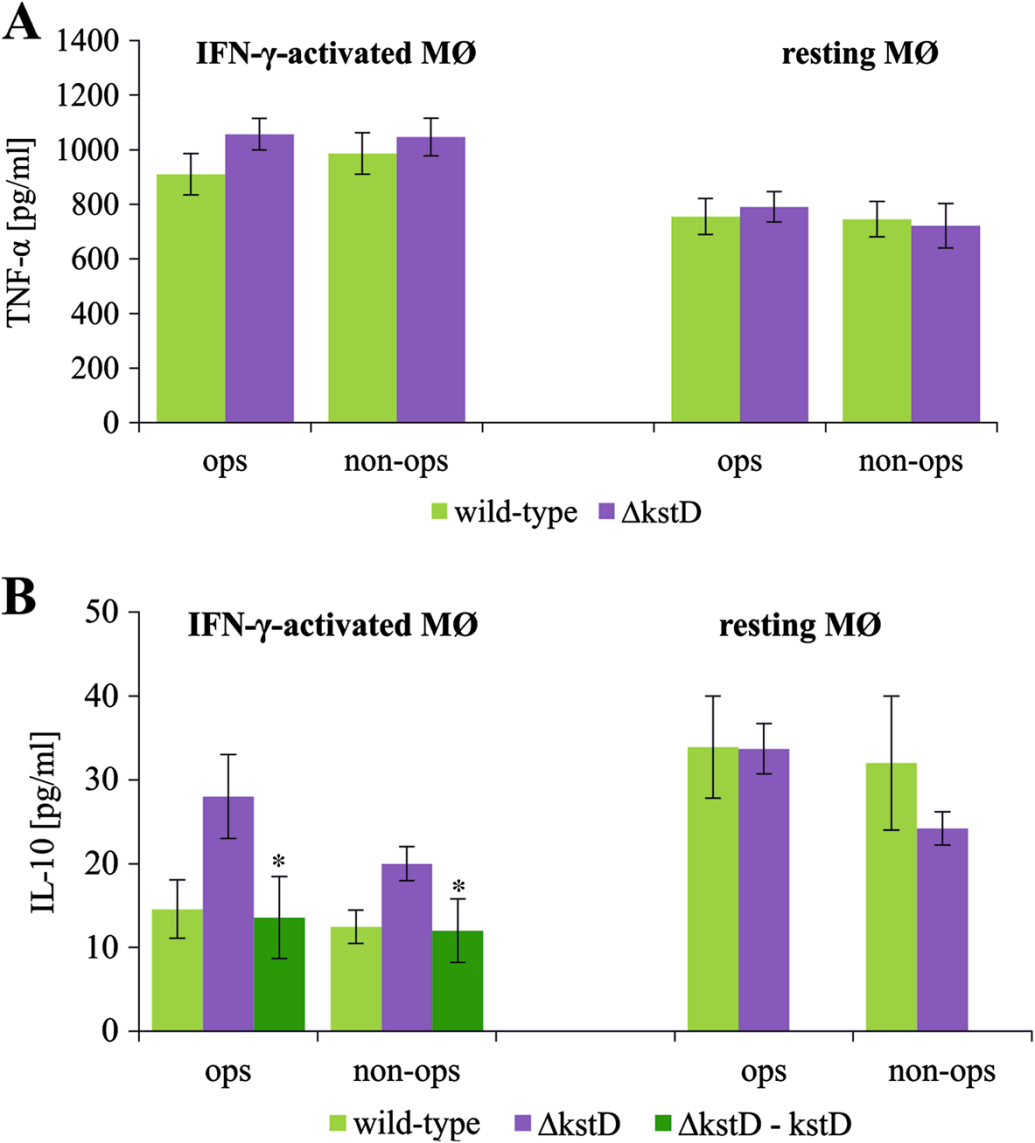
**TNF-α and IL-10 production by infected MØ.** Resting MØ and IFN-γ-activated MØ were infected with wild-type, ∆*kstD*, or ∆*kstD*-*kstD* strains for 2 hours and then cultured for 1 day. The amount of released TNF-α (**A**) and IL-10 (**B**) was assessed in culture supernatants using ELISA kits. Data are presented as means (pg/ml) ± SEMs (n = 5; *p ≤ 0.02, ∆*kstD* vs. wild-type or ∆*kstD*-*kstD*; Mann–Whitney *U* test). ops – bacteria opsonized, non-ops – bacteria non-opsonized.

We found no difference in the production of TNF-α between resting and IFN-γ-activated MØ infected with either wild-type or mutant strains (Figure 6A). Similarly, resting MØ produced equal amounts of IL-10 in response to the infection with wild-type Mtb or Δ*kstD* strain. However, the Δ*kstD* strain, both opsonized and non-opsonized, stimulated IFN-γ-activated MØ to release significantly higher amounts of IL-10 (20 ± 2 and 28 ± 6 pg/ml, respectively) than did wild-type (13 ± 2 and 15 ± 4 pg/ml, respectively) or complemented strains (12 ± 4 and 14 ± 5 pg/ml, respectively) (Figure 6B). Furthermore, resting MØ infected with wild-type Mtb produced higher amounts of IL-10 than did IFN-γ-activated MØ. In the absence of Mtb infection, resting and IFN-γ-activated MØ released relatively low amounts of TNF-α (11.0 ± 3.0 and 8.2 ± 2.2 pg/ml for resting and activated MØ, respectively) and IL-10 (1.3 ± 0.4 and 2.8 ± 0.3 pg/ml for resting and activated MØ, respectively).

## Discussion

It is well documented that Mtb metabolizes cholesterol, though the role of this metabolism in pathogenicity remains unclear. Various Mtb mutants defective in the ability to transport or degrade cholesterol have been previously investigated in respect to possible attenuation of the infection process. The deletion of *mce4*, which affects cholesterol uptake by Mtb, attenuated the Δ*mce4* mutant in IFN-γ-stimulated, but not resting, mouse bone marrow MØ [9]. Disruption of the gene encoding 3-ketosteroid 9α-hydrolase attenuated the growth of *ΔkshA* and *ΔkshB* mutants in both resting and IFN-γ-activated, mouse bone marrow MØ [11]. The inhibition of side chain degradation by inactivation of *fadA5* decreased the virulence of mutant during the late stage of mouse infection [19]. It was also previously shown that Δ*igr* knock-out strain of Mtb was attenuated in mice during the early phase of infection [20]. The *igr* of Mtb was identified as required for degradation of the 26-propionate side chain fragment [21,22]. The above data suggest that ability of Mtb to catabolize cholesterol is important during both early and late stages of the infection. In contrast, Yang et al. [23] reported that replication rates of wild-type Mtb CDC1551 and its mutant ∆*hsd* were similar in the lungs of guinea pigs and concluded that cholesterol was not an essential source of nutrient for Mtb during infection. On the other hand, Mtb H37Rv ∆*hsd* mutant (as well as double mutant ∆*hsd*∆*choD*) were able to utilize cholesterol suggesting that both HsdD and ChoD are not essential for cholesterol degradation [13]. All above-mentioned examples described the activity of Mtb mutants in animal models. However, the intracellular replication of mutants defective in the ability to degrade cholesterol and their effects on the functional activity of human MØ are less well understood. Therefore, the aim of our study was to determine whether the ∆*kstD* mutant can multiply in human MØ and assess its capacity to modify the functional activity of the phagocytes. As we demonstrated previously, KstD is an essential enzyme in the metabolism of cholesterol by Mtb; therefore, the ∆*kstD* strain is unable to use cholesterol as a primary source of carbon and energy, and accumulates the non-toxic derivatives of cholesterol, AD and 9OHAD. Moreover, the *in vitro* growth of ∆*kstD* strain is not affected in rich medium compared to the wild type [10].

Herein, we found that the lack of a functional *kstD* gene did not influence the ability of resting or IFN-γ-activated MØ to ingest Mtb. However, we observed that the intracellular replication of ∆*kstD* mutant was attenuated in both resting (statistically significant) and IFN-γ-activated (statistically insignificant) MØ compared to the wild-type strain. The attenuation of cholesterol degradation mutants was previously observed in IFN-γ-activated MØ [9,11]. Our data suggest that cholesterol degradation ability is important for Mtb at multiple stages of the infection in resting and IFN-γ-activated MØ. The significant attenuation of the mutant observed in our study in resting MØ may result from experimental model used - human cell line THP-1.

Tubercle bacilli after penetration into MØ reside predominantly in a cholesterol rich region of cell plasma membrane and the ability of Mtb to degrade cholesterol would give tubercle bacilli an advantage within the host. However, it is unclear whether the only reason of attenuation of cholesterol degradation mutants in MØ is due to their inability to use cholesterol as a source of carbon and energy. It was previously found that a mutant lacking an intact *hsaC* gene accumulated catechol derivatives that appeared to be toxic to Mtb [12]. The attenuated growth of the ∆*kstD* mutant in resting MØ, used in the current study, was not due to the accumulation of toxic compounds, suggesting that cholesterol degradation ability *per se* is essential for the replication of tubercle bacilli inside MØ [10]. On the other hand, the lack of a functional copy of *kstD* might modify the basic metabolism affecting pathogenic features of the bacilli. The mutant *ΔkshB* revealed unusual change in the structure of the cell wall which was thickened and loosened as a result of the synthesis of lipid types other than those in wild-type Mtb [11]. Such modification of the cell envelope can influence the pathogenicity of Mtb.

It was also suggested that cholesterol metabolism of Mtb may contribute to the production of specific virulence factors and/or disruption of host cell signaling [24].

Moreover, the *in vivo* cholesterol degradation by Mtb can affect the activity of MØ. In our studies the ∆*kstD* failed to inhibit ROS and NO production in resting MØ compared to wild-type and complemented strains. It is generally accepted that ROS and RNIs kill or inhibit intracellular growth of Mtb [8,25,26]. Similar to previous report [27], we found that Mtb induced ROS production in MØ immediately after phagocytosis (data not shown). The increased oxidative response in MØ infected with ∆*kstD* unable to metabolize cholesterol can be directly related to cholesterol degradation process (e.g. if cholesterol metabolite modifies the signaling of enzymes involved in NO and ROS production) or can be a derivative of attenuation of bacilli inside MØ. To clarify this issue we used two different Mtb mutants, not related to cholesterol degradation process and showing attenuated growth in THP-1, to test them in respect to inhibition of ROS/NO production in macrophages (data not shown). Only one of them was able to inhibit ROS/NO production to the level of the wild type strain. Therefore the most likely interpretation of our result is that ROS/NO over-production in resting MØ infected with Δ*kstD* results from the attenuation of the mutant’s growth inside MØ, however the specific role of cholesterol degradation intermediates cannot be excluded. Changes in the cholesterol level in plasma membrane modulate the activity of the proteins and the receptors located in the lipid rafts. The components of NADPH oxidase are known to migrate to the plasma membrane of newly formed phagosome. The recruitment of NADPH oxidase subunits and their assembly in the membrane are necessary for an oxidative burst execution [28]. It was found that methyl-β-cyclodextrin, a chelating agent of cholesterol, inhibited H_2_O_2_ production by neutrophils and in the absence of lipid rafts neutrophil NADPH oxidase activity was changed [29].

We did not observe differences in oxidative response in IFN-γ induced MØ infected with wild type and mutant strains. However, the IFN-γ induces iNOS expression initiating the production of NO by MØ prior to their infection with Mtb (data not shown). The high level of NO reached in IFN-γ treated MØ cannot be subsequently lowered even by wild type Mtb at least within the period of the experiment. Therefore, IFN-γ-activated MØ produced a similar, high amount of NO in response to the infection with wild-type or mutant strains.

Phagocytosis of Mtb initiates the production of both TNF-α and IL-10 by MØ. It has been demonstrated by others that TNF-α together with IFN-γ participate in the killing of Mtb through the induction of NO and ROS production. TNF-α is also essential for granuloma formation [30-32]. We found here that the infection of resting and INF-γ-activated MØ with wild-type Mtb or *ΔkstD* mutant caused the release of equal amounts of TNF-α. At the same time however, we observed a greater increase in the production of IL-10 by IFN-γ-activated MØ infected with the *ΔkstD* strain compared to those infected with the wild-type or complemented strains. It has been reported that pathogenic strains of Mtb stimulate lower levels of TNF-α production by MØ than non-pathogenic species [32]. IL-10 is an immunosuppressive cytokine that blocks phagosome maturation and antigen presentation and also limits the Th1 response [33]. Thus, our finding that MØ infected with the *ΔkstD* strain produce higher level of IL-10 than MØ infected with wild-type Mtb and that similar amount of TNF-α is released by MØ after infection with both strains may suggest that certain aspects of the virulence activity of the wild-type strain are in fact not affected in the *ΔkstD* mutant.

Interestingly, we found that blocking the TLR2-mediated signaling pathway prior to infection restored the phenotype of the *ΔkstD* mutant in resting MØ to a level similar to that of the wild-type strain. However, neither anti-TLR2 blocking mAb nor IRAK1/4 inhibitor altered the response of MØ to wild-type Mtb. These results suggest that TLR2 signaling is disrupted in MØ infected with wild-type Mtb*,* but not in MØ infected with the mutant strain. The essential role of the TLR2-mediated pathway in the production of NO and ROS in Mtb-infected MØ is well documented [5,6,26,34]. Further study is needed to elucidate the complete mechanism by which Mtb affects TLR2 signaling whether the ability of Mtb to catabolize cholesterol might be important for this process. It has been demonstrated by others that Mtb is able to modulate macrophage signaling pathways by stimulating phosphorylation of the Bcl-2 family member Bad as well as AKT kinase [35]. AKT kinase serves a pro-survival function by inhibiting apoptotic processes [36], whereas Bad is a pro-apoptotic member of Bcl-2 family involved in initiating apoptosis [37].

## Conclusions

Our results indicate that the degradation of cholesterol is required for Mtb to survive during infection in resting macrophages. A mutant lacking a functional copy of the *kstD* gene showed a limited ability to multiply inside resting MØ. Moreover, the bactericidal activity of resting MØ was not inhibited by the infection with the *ΔkstD* mutant strain. Collectively, these findings indicate a relationship between degradation of cholesterol by Mtb, Mtb survival in MØ, and functional responses of Mtb-infected MØ.

## Competing interests

The authors report no conflicts of interests.

## Authors’ contributions

MB, IS, MiK, AB, and JP carried out the experiments and participated in the interpretation, acquisition, and statistical analysis of data. MaK and JD made substantial contributions to the conception and design of the study as well as to interpretation of study results. MaK, JD, and ZS were involved in drafting and critical revisions of the manuscript, and gave final approval of the version to be published. All authors have read and approved the final manuscript.
